# Acute hemorrhagic encephalitis in a pregnant woman with coronavirus disease-2019

**DOI:** 10.1590/0037-8682-0630-2021

**Published:** 2022-04-08

**Authors:** Rojbin Ceylan Tekin, Yurdagül Tolu Gökhaner, Recep Tekin

**Affiliations:** 1 Mardin State Hospital, Department of Radiology, Diyarbakir, Turkey.; 2 Dicle University, Faculty of Medicine, Department of Infectious Diseases and Clinical Microbiology, Diyarbakir, Turkey.

**FIGURE 1:**
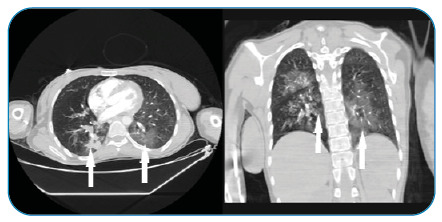
The unenhanced axial and coronal reformatted thorax CT images show peribronchovascular and subpleural ground-glass opacities and vascular dilatation (arrows) in the bilateral lung.

**FIGURE 2:**
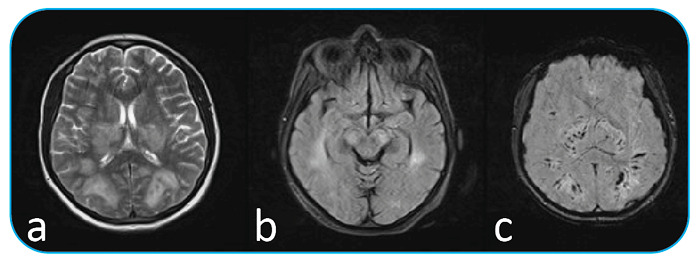
MRI brain images with bilateral periventricular, thalamic, parahippocampal, and mesencephalic lesions shown with hyperintensity in T2WI **(a)** and FLAİR **(b)** Internal hemorrhage causing susceptibility changes in SWI images **(c)**.

A 32-year-old pregnant woman with a 12-day history of fever and cough was admitted to the emergency department for confusion and severe headache at 24 weeks of gestation. She reported a worsening condition for 5 days with headache, confusion, and dyspnea. Neurological examination showed dysarthria and mild hemihypoesthesia. A polymerase chain reaction test from the nasopharyngeal swab confirmed the diagnosis of coronavirus disease (COVID-19). Chest computed tomography (CT) scan was also highly suggestive of COVID-19. The unenhanced axial and coronal reformatted thorax CT image demonstrates peribronchovascular and subpleural ground-glass opacities and vascular dilatation (arrows) in the bilateral lung (Figure 1). Magnetic resonance imaging (MRI) of the brain showed bilateral periventricular, thalamic, parahippocampal, and mesencephalic lesions with hyperintensity in FLAİR and T2WI, internal hemorrhage that caused susceptibility changes in SWI images (Figure 2). In this case, the baby was delivered by cesarean section. The patient developed hypoxic respiratory failure and progressed to severe acute respiratory distress syndrome on day 12 and required intubation and mechanical ventilation under heavy sedation. Pregnant women with COVID-19 are more likely to have a preterm birth. Acute hemorrhagic encephalitis (AHE) is a rare and often fatal neurological complication of COVID-19^1^ ^,^ ^2^. AHE should be considered in the differential diagnosis of patients presenting with neurological symptoms in patients with COVID-19^3^. High clinical suspicion and early imaging diagnosis of this condition can enable clinicians to pursue more aggressive treatment options to reduce fatal outcomes.
